# Diagnostic Performance of Articular Ultrasound Versus Magnetic Resonance Imaging in the Approach to Rotator Cuff Lesions at a Referral Hospital

**DOI:** 10.7759/cureus.81149

**Published:** 2025-03-25

**Authors:** Daniel F Duque, Jose Luis Montoya Restrepo, Juan D Ayala Torres, Juan Llano, Amalia Patiño Rengifo

**Affiliations:** 1 Radiology, Servicios de Salud San Vicente Fundación, Medellín, COL; 2 Radiology, Universidad de Antioquia, Medellín, COL; 3 Radiology, Clínica Las Américas Auna, Medellín, COL

**Keywords:** arthroscopy, diagnostic imaging modalities, magnetic resonance imaging, magnetic resonance (mr), rotator cuff injuries, ultrasonography (us)

## Abstract

Introduction

Rotator cuff injury is a leading cause of musculoskeletal pain and the most common shoulder pathology. It is characterized by pain and limited mobility, particularly during overhead activities, and is associated with factors such as age, trauma, occupation, limb dominance, and cardiovascular risk. Diagnosis requires clinical evaluation and imaging, with ultrasound being a cost-effective and accessible option, whereas MRI is preferred for persistent or ambiguous cases. However, MRI access may be restricted by geographical and economic constraints.

Methodology

A retrospective census was conducted at a fourth-level hospital in Medellín, Antioquia (2015-2020), following research committee approval. Patients over 18 years old with suspected rotator cuff injury who underwent arthroscopy and had MRI and ultrasound reports within six months were included. Imaging and surgical data were retrieved from Xen RIS HIRUKO and Matrix systems.

Ultrasound was performed using a General Electric Logiq S8 (15 MHz transducer) (General Electric Healthcare, Chicago, IL) and a Toshiba Aplio 400 (18 MHz transducer) (Toshiba Medical Systems Corporation, Tokyo, Japan). MRI was conducted on a Philips Achieva 1.5 T (Philips Healthcare, Eindhoven, Netherlands) with standard musculoskeletal protocols. Data were collected independently, processed in Excel (Microsoft Corporation, Redmond, WA), and analyzed using STATA 14 (StataCorp LLC, College Station, TX). Quantitative variables were reported as means and standard deviations, with normality assessed using the Kolmogorov-Smirnov test, while qualitative variables were expressed as frequencies.

Results

Twenty-four patients (62.4 ± 12.2 years) had 27 rotator cuff injuries; eight (33.3%) were traumatic (all men), and 16 (66.7%) were degenerative. Right-sided lesions occurred in 12 (50%), left-sided in nine (37.5%), and bilateral in three (12.5%). The most common arthroscopic finding was a complete supraspinatus rupture in 16 shoulders (59%), followed by subscapularis partial ruptures not exceeding 50% in 10 shoulders (37%) and supraspinatus partial ruptures exceeding 50% in seven shoulders (26%).

For supraspinatus evaluation, ultrasound detected 79% of lesions and MRI detected 75%, both achieving 93.7% sensitivity for complete ruptures. For partial ruptures exceeding 50%, both modalities demonstrated good specificity and predictive values but low sensitivity (42.8%), with an area under the curve (AUC) of 0.78 for complete ruptures. Regarding the subscapularis, MRI detected 54.5% of lesions compared to 26.3% detected by ultrasound, demonstrating higher specificity. In the infraspinatus, both modalities detected only one of two complete ruptures, with several false positives. Agreement with arthroscopy was moderate for the supraspinatus (kappa: ultrasound = 0.59; MRI = 0.448) and low for the subscapularis (kappa: ultrasound = 0.25; MRI = 0.56), with ultrasound performing better in traumatic cases.

Conclusions

This study provides a direct comparison between ultrasound and MRI in the evaluation of rotator cuff injuries, with arthroscopy as the reference standard. While both modalities were effective in detecting complete supraspinatus tears, MRI demonstrated greater accuracy for subscapularis tendon assessment. These findings support the continued use of ultrasound as a practical and reliable tool, particularly in resource-limited settings.

## Introduction

Rotator cuff injury is a leading cause of musculoskeletal pain and the most common shoulder pathology [1,2]. It is characterized by pain and reduced mobility during overhead activities [2] and is strongly associated with age [1,3,4], as well as trauma history, occupation, limb dominance, and cardiovascular risk factors [4,5].

This condition contributes to disability and high healthcare costs, with a prevalence of 22.1% in the general population and no significant differences between sexes [3]. Prevalence increases with age, affecting approximately 9.7% of individuals at 20 years old compared to 62% of those over 80 years old, and remains high even in asymptomatic cases [6]. Clinical guidelines recommend integrating imaging findings with clinical examination for diagnosis [7].

Evidence supports the use of ultrasound (US) and magnetic resonance imaging (MRI) for evaluating the rotator cuff [7]. Ultrasound is suggested for ruling out tears when conservative management fails or an MRI is unavailable, whereas MRI is recommended for prolonged or unexplained pain, shoulder weakness, or inconclusive ultrasound findings [8].

Although arthroscopy is the gold standard for diagnosing shoulder pathologies, its invasiveness, and associated risks make MRI the preferred pre-surgical imaging modality [9,10]. However, in our setting, access to MRI is often limited due to geographical barriers and constraints within the social health security system.

Ultrasound has emerged as a viable, cost-effective alternative to MRI due to advancements in transducer power, resolution, and operator training [11-13]. It provides a non-invasive imaging option, reducing waiting times and facilitating early diagnosis.

This study aimed to assess the diagnostic accuracy of ultrasound and standard shoulder MRI in detecting tendinosis, partial tears, and complete rotator cuff tendon tears, using arthroscopy as the reference standard.

## Materials and methods

**Figure 1:**
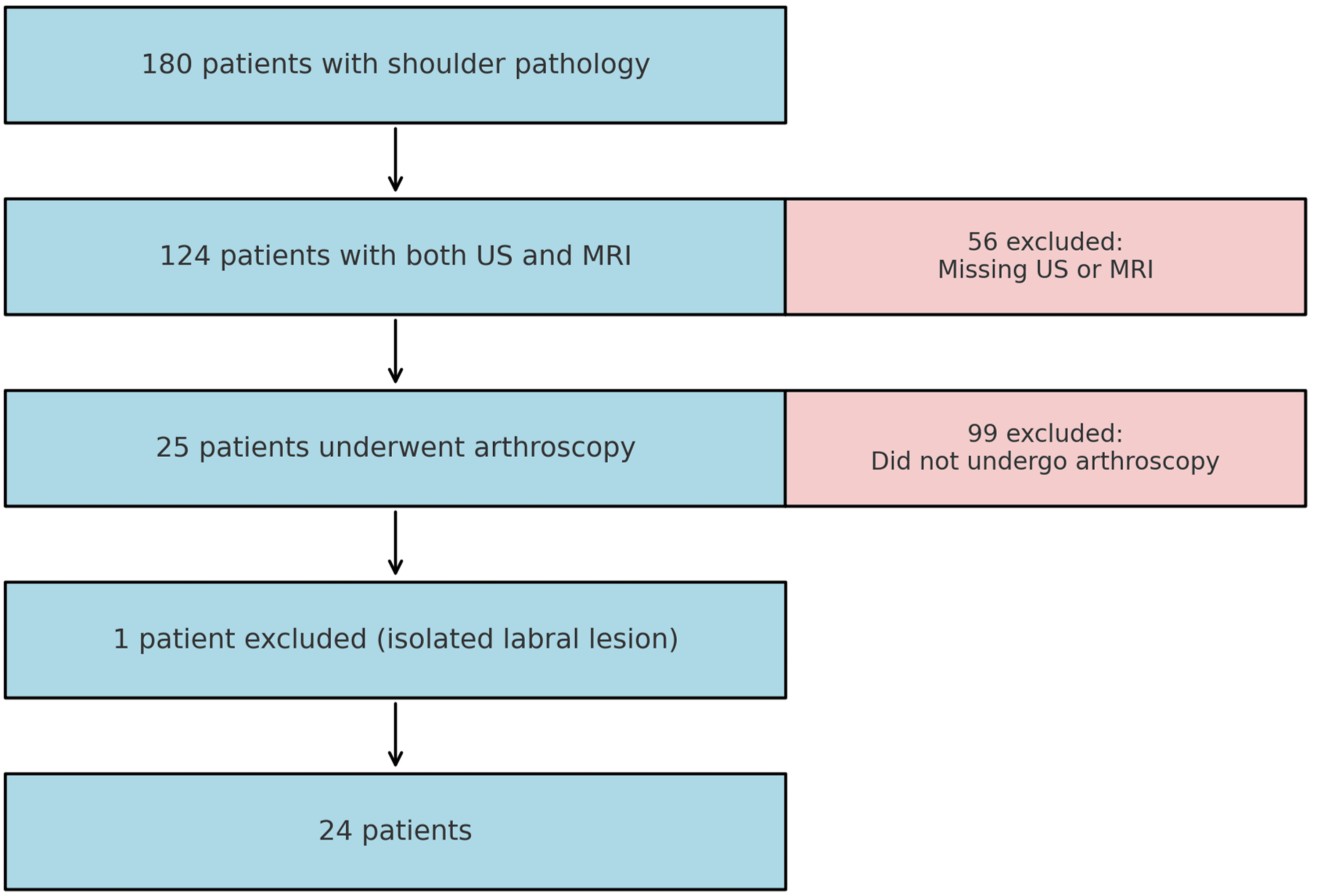
Flowchart of patient selection and exclusion criteria. US: ultrasound; MRI: magnetic resonance imaging.

Following approval from the research committee, a retrospective census was conducted at a fourth-level hospital in Medellín, Antioquia, covering the period from January 2015 to December 2020. Patients over 18 years of age with suspected rotator cuff injury who underwent arthroscopy and had both MRI and ultrasound performed at the same institution, within a maximum interval of six months, were included. A database of 180 patients with shoulder pathology was compiled, of whom 124 had reports from both ultrasound and MRI. However, only 25 patients underwent arthroscopy, three of whom presented with bilateral lesions. One patient with an isolated labral lesion was excluded, resulting in a final study population of 24 patients and a total of 27 shoulders analyzed (Figure 1).

Most ultrasound examinations were performed by a radiologist specializing in musculoskeletal imaging; no differential analysis was conducted for ultrasounds performed by general radiologists. Imaging was conducted using a General Electric Logiq S8 (15 MHz transducer) (General Electric Healthcare, Chicago, IL) and a Toshiba Aplio 400 (18 MHz transducer) (Toshiba Medical Systems Corporation, Tokyo, Japan). MRI scans were interpreted by a musculoskeletal radiologist and performed on a Philips Achieva 1.5 T (Philips Healthcare, Eindhoven, Netherlands), using a protocol comprising proton density-weighted sequences (axial and coronal), short tau inversion recovery (STIR) sequences (coronal and sagittal), and spectral attenuated inversion recovery (SPAIR) and T1 spin-echo sequences (axial).

Data were obtained from imaging reports and surgical descriptions extracted from the Xen RIS HIRUKO and Matrix systems. Two independent investigators collected and processed the data in an Excel spreadsheet (Microsoft Corporation, Redmond, WA). The data were then analyzed using STATA 14 (StataCorp LLC, College Station, TX). Quantitative variables were expressed as means and standard deviations, with normality assessed using the Kolmogorov-Smirnov test, while qualitative variables were reported as absolute numbers and frequencies. Sensitivity, specificity, positive predictive value (PPV), negative predictive value (NPV), and the area under the curve (AUC) were calculated using arthroscopy as the gold standard. A 95% confidence interval was applied. Agreement between diagnostic methods and arthroscopy was assessed using the kappa coefficient, with further stratification by etiology (traumatic vs. degenerative).

## Results

Twenty-four patients were included in the study, contributing to the analysis of 27 shoulders affected by rotator cuff injuries. The mean age was 62.4 years (SD: 12.2), with a predominance of male patients (13; 54.2%). Twelve patients (50%) had right-sided lesions, nine (37.5%) had left-sided lesions, and three (12.5%) had bilateral involvement. The traumatic etiology was identified in eight patients (33.3%), all men, while the remaining 16 (66.7%) had degenerative etiology, the only cause of injury in women. Among men, six left and six right shoulders were affected, with one bilateral case, whereas among women, four left and nine right shoulders were affected, with two bilateral cases (Table 1).

**Table 1:** Demographic and clinical characteristics of the study population.

Variable	Total (n = 24)	Male (n = 13)	Female (n = 11)
Age (mean ± SD)	62.4 ± 12.2	-	-
Laterality			
Right	12 (50.0%)	6 (46.2%)	6 (54.5%)
Left	9 (37.5%)	6 (46.2%)	3 (27.3%)
Bilateral	3 (12.5%)	1 (7.7%)	2 (18.2%)
Etiology			
Traumatic	8 (33.3%)	8 (61.5%)	0 (0.0%)
Degenerative	16 (66.7%)	5 (38.5%)	11 (100%)

The most common arthroscopic finding was a complete rupture of the supraspinatus tendon in 16 shoulders (59%). Additionally, seven cases (26%) presented partial ruptures exceeding 50%, one case (4%) had tendinosis, and three cases (11.11%) showed no abnormalities. The subscapularis tendon was the second most affected, with 10 shoulders (37%) presenting partial ruptures of less than 50% and one case (4%) of tendinosis (Table 2).

**Table 2:** Findings detected by each diagnostic method.

Diagnostic method					
Arthroscopic					
Tendon	No findings	Tendinosis	Partial < 50%	Partial > 50%	Total rupture
Supraspinatus	3 (11.11%)	1 (4%)	0 (0%)	7 (26%)	16 (59%)
Subscapularis	16 (59%)	1 (4%)	10 (37%)	0 (0%)	0 (0%)
Infraspinatus	25 (93%)	0 (0%)	0 (0%)	0 (0%)	2 (7%)
Biceps	26 (96%)	1 (4%)	0 (0%)	0 (0%)	0 (0%)
Ultrasound					
Supraspinatus	2 (7.41%)	1 (3.7%)	2 (7.41%)	3 (11.11%)	19 (70.37%)
Subscapularis	13 (48.15%)	5 (18.52%)	8 (29.63%)	1 (3.7%)	0 (0%)
Infraspinatus	21 (77.78%)	2 (7.41%)	3 (11.11%)	0 (0%)	1 (3.7%)
Biceps	25 (92.59%)	2 (7.41%)	0 (0%)	0 (0%)	0 (0%)
Magnetic resonance					
Supraspinatus	3 (11.11%)	1 (3.7%)	0 (0%)	4 (14.81%)	19 (70.37%)
Subscapularis	19 (70.37%)	2 (7.41%)	5 (18.52%)	1 (3.7%)	0 (0%)
Infraspinatus	19 (70.37%)	0 (0%)	7 (25.93%)	0 (0%)	1 (3.7%)
Biceps	27 (100%)	0 (0%)	0 (0%)	0 (0%)	0 (0%)

**Figure 2:**
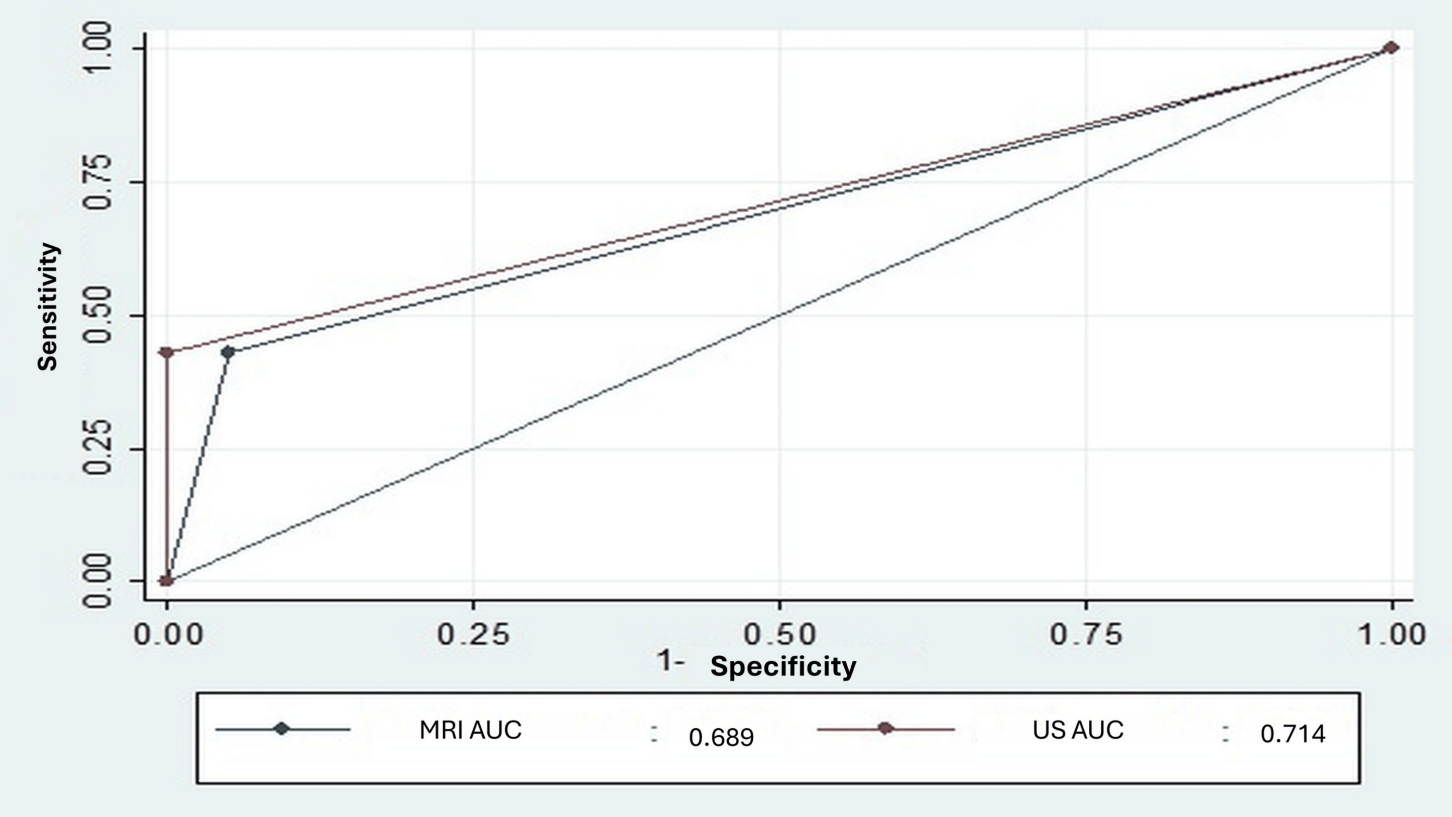
Area under the curve for ultrasound and MRI in partial rupture of the supraspinatus greater than 50%. MRI: magnetic resonance imaging; US: ultrasound; AUC: area under the curve.

**Figure 3:**
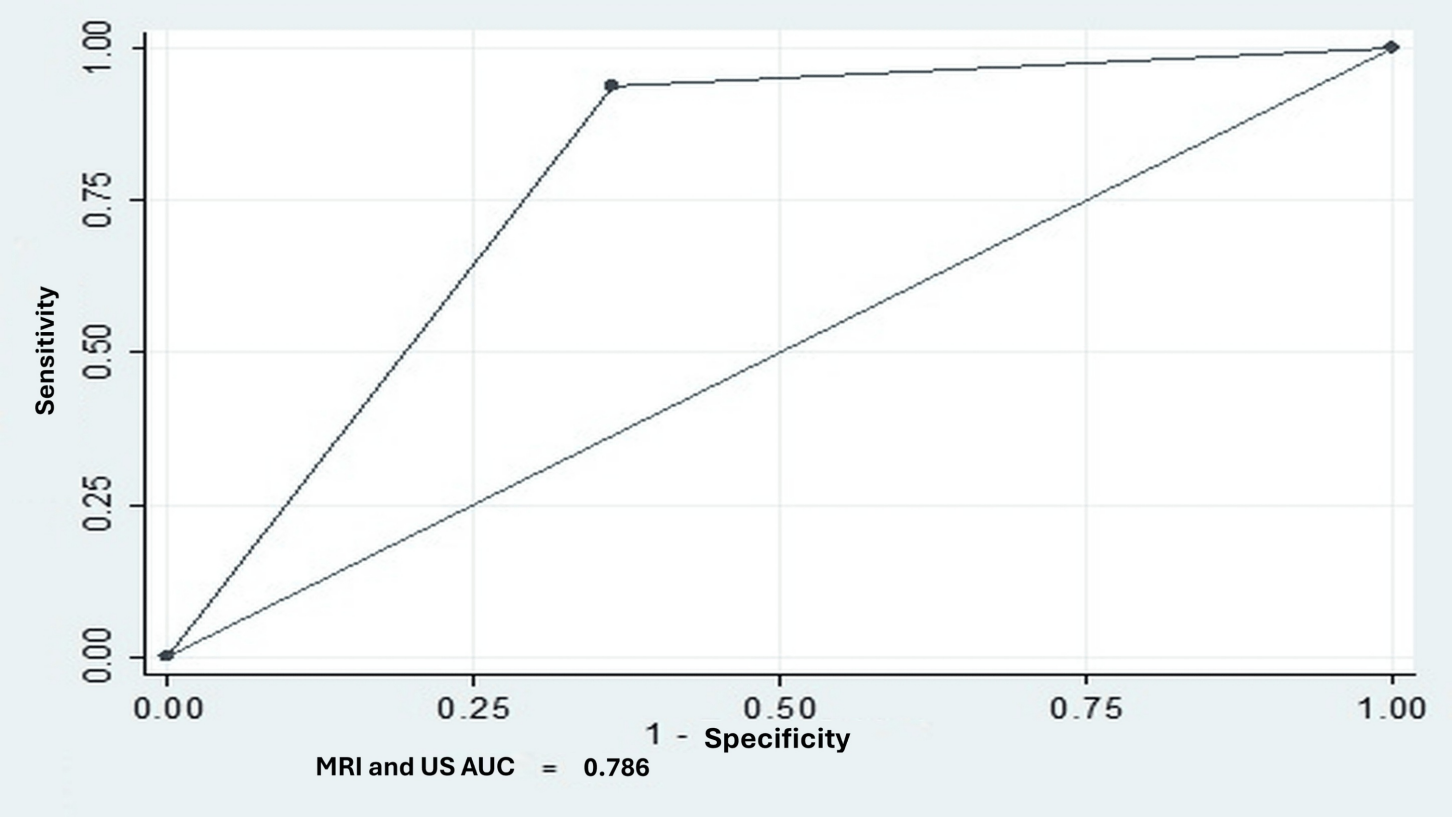
Area under the curve for ultrasound and MRI in total rupture of the supraspinatus. MRI: magnetic resonance imaging; US: ultrasound; AUC: area under the curve.

For supraspinatus evaluation, ultrasound detected 79% of lesions, while MRI detected 75%. Both methods correctly identified 15 out of 16 complete ruptures, achieving a sensitivity of 93.7%. For partial ruptures greater than 50%, both techniques demonstrated good specificity and predictive values, although sensitivity was low (42.8%) (Table 3). The predictive capability, measured by the area under the curve (AUC), was 0.78 for complete ruptures and moderate for partial ruptures greater than 50% (Figures 2, 3).

**Table 3:** Diagnostic accuracy of ultrasound and MRI compared to arthroscopy for the supraspinatus tendon. CI: confidence interval; PPV: positive predictive value; NPV: negative predictive value; AUC: area under the curve. * Interval not calculable by STATA.

Diagnostic method				
Ultrasound				
	Tendinosis	Partial < 50%	Partial > 50%	Total rupture
Sensitivity	100% (CI 20.65 - 100)	-	42.86% (CI 15.82 - 74.95)	93.75% (CI 71.67 - 98.89)
Specificity	100% (CI 87.13 - 100)	92.59% (CI 76.63 - 97.94)	100% (CI 83.89 - 100)	63.64% (CI 35.38 - 84.83)
PPV	100% (CI 20.65 - 100)	0.0% (CI 0.0 - 65.76)	100% (CI 43.85 - 100)	78.95% (CI 56.67 - 91.49)
NPV	100% (CI 87.13 - 100)	100% (CI 86.68 - 100)	83.33% (CI 64.15 - 93.32)	87.5% (CI 52.91 - 97.76)
AUC	1.00*	-	0.71 (CI 0.51 - 0.91)	0.78 (CI 0.62 - 0.94)
MRI				
Sensitivity	0.0% (CI 0.0 - 79.35)	-	42.86% (CI 15.82 - 74.95)	93.75% (CI 71.67 - 98.89)
Specificity	88.46% (CI 71.02 - 96.0)	-	95% (CI 76.39 - 99.11)	63.64% (CI 35.38 - 84.83)
PPV	0.0% (CI 0.0 - 56.15)	-	75% (CI 30.06 - 95.44)	78.95% (CI 56.67 - 91.49)
NPV	95.83% (CI 79.76 - 99.26)	-	82.61% (CI 62.86 - 93.02)	87.5% (CI 52.91 - 97.76)
AUC	0.44*	-	0.68 (CI 0.48 - 0.89)	0.78 (CI 0.62 - 0.94)

**Figure 4:**
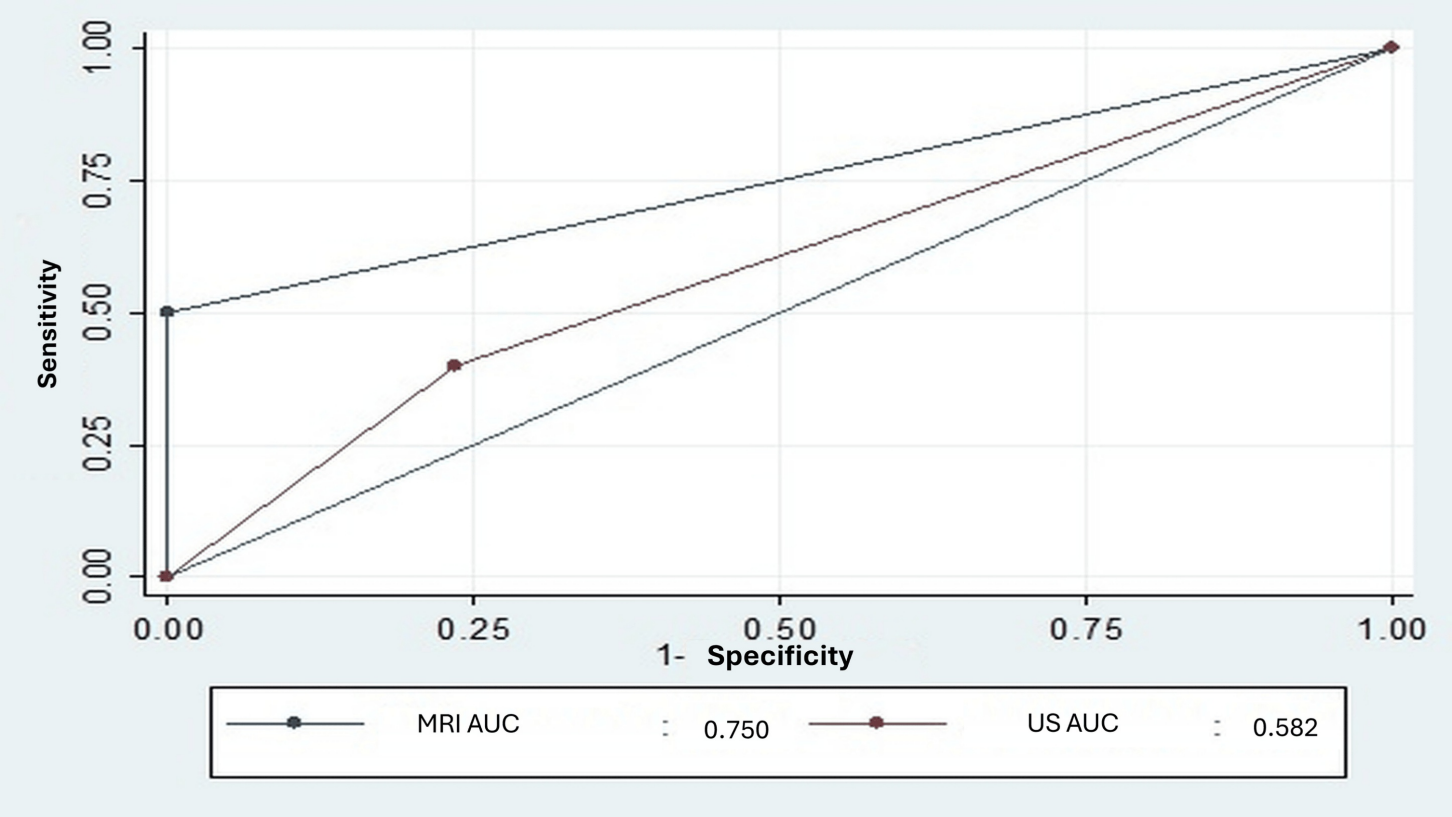
Area under the curve for ultrasound and MRI in partial rupture of less than 50% of the subscapularis. MRI: magnetic resonance imaging; US: ultrasound; AUC: area under the curve.

Regarding the subscapularis tendon, ultrasound correctly identified 26.3% of cases, compared to 54.5% with MRI. The low number of true positives reduced sensitivity; however, MRI demonstrated superior specificity (96.1% for tendinosis and 100% for partial ruptures <50%) compared to ultrasound (80% and 76.7%, respectively) (Table 4). Ultrasound exhibited poor discriminatory ability for this tendon, with AUC values below 0.6 (Figure 4).

**Table 4:** Diagnostic accuracy of ultrasound and MRI compared to arthroscopy for the subscapularis tendon. CI: confidence interval; PPV: positive predictive value; NPV: negative predictive value; AUC: area under the curve. * Interval not calculable by STATA.

Diagnostic method			
Ultrasound			
	Tendinosis	Partial < 50%	Partial > 50%
Sensitivity	0.0% (CI 0.0 - 79.35)	40% (CI 16.82 - 68.73)	-
Specificity	80.77% (CI 62.12 - 91.49)	76.47% (CI 52.74 - 90.45)	96.3% (81.72 - 99.34)
PPV	0.0% (CI 0.0 - 43.45)	50% (CI 21.52 - 78.48)	0.0% (0.0 - 79.35)
NPV	95.45% (CI 78.2 - 99.19)	68.42% (CI 46.01 - 84.64)	100% (87.13 - 100)
AUC	0.4*	0.58 (CI 0.39 - 0.77)	-
MRI			
Sensitivity	100% (20.65 - 100)	50% (23.66 - 76.34)	-
Specificity	96.15% (81.11 - 99.32)	100% (81.57 - 100)	96.3% (81.72 - 99.34)
PPV	50% (9.453 - 90.55)	100% (56.55 - 100)	0.0% (0.0 - 79.35)
NPV	100% (86.68 - 100)	77.27% (56.56 - 89.88)	100% (87.13 - 100)
AUC	0.98*	0.75 (CI 0.58 - 0.91)	-

For infraspinatus injuries, both imaging methods detected only one of two complete ruptures, with five false positives on ultrasound and seven on MRI (Table 2). Diagnostic precision for the infraspinatus and biceps was not evaluated due to the low prevalence of lesions.

The agreement between both methods and arthroscopy was moderate for the supraspinatus, with a kappa coefficient of 0.59 (p < 0.001) for ultrasound and 0.448 (p < 0.001) for MRI. When stratified by etiology, ultrasound agreement was higher in traumatic cases (k = 0.84) and lower in degenerative cases (k = 0.38), whereas MRI showed consistent agreement values. For the subscapularis tendon, ultrasound exhibited low concordance with arthroscopy (k = 0.25, p = 0.62) compared to MRI (k = 0.56, p < 0.001). Ultrasound agreement was higher in traumatic cases (k = 0.37) but lower in degenerative cases (k = 0.19), whereas MRI maintained similar values for both traumatic (k = 0.50) and degenerative cases (k = 0.60).

## Discussion

The findings of this study indicate that both ultrasound and MRI exhibit high sensitivity and negative predictive value for detecting complete supraspinatus tears, though their diagnostic performance is lower for partial tears. The concordance of both methods with arthroscopy is moderate, with MRI demonstrating superior diagnostic accuracy and agreement in evaluating the subscapularis tendon compared to ultrasound.

The most common arthroscopic finding was a complete supraspinatus tear, aligning with existing literature that highlights the strong diagnostic performance of both imaging modalities for these lesions [9,12,14,15]. The high prevalence of complete tears and the operator-dependent nature of ultrasound [14] may explain the variability in findings across studies [9,12,16]. Partial tears remain challenging to assess; in our sample, both modalities demonstrated low sensitivity but good specificity, with detection rates comparable to previous studies [12,15]. Although ultrasound appeared to enhance the detection of tendinosis, the presence of only one case in our sample limits definitive conclusions regarding its superiority over MRI.

Some studies use MRI as the reference standard rather than arthroscopy, potentially affecting MRI specificity, as certain intrasubstance tears detected by MRI may not be surgically confirmed [16]. Additionally, interobserver variability in arthroscopy can impact the assessment of partial tears [17].

For the subscapularis tendon, partial tears < 50% were most prevalent, and MRI exhibited greater specificity than ultrasound, consistent with the findings of Farooqi et al. [12] and Adams et al. [18]. This may be attributed to ultrasound’s limited access to the tendon [19] and its consistently low sensitivity [12,13]. MRI is thought to provide a more accurate assessment of lesion location and extent, particularly for evaluating fatty infiltration and atrophy [13,14], given that most subscapularis tears originate intra-articularly [20].

Ultrasound has shown comparable performance to MRI in the evaluation of the supraspinatus tendon, likely due to advancements in imaging technology and improved operator expertise, both of which have contributed to increased diagnostic accuracy [12,13,21]. Nevertheless, ultrasound still exhibits substantial interobserver variability [22]. Notably, this variability tends to be lower in trauma-related cases [23], whereas MRI interpretations do not show a similar pattern of agreement.

Both ultrasound and MRI are considered cost-effective imaging modalities [24]. However, in our setting, ultrasound offers distinct advantages in terms of lower cost and greater accessibility. Therefore, when factors such as budget constraints, safety, radiologist expertise, or equipment availability limit the use of MRI, ultrasound becomes the preferred alternative [14,16,24,25].

This study has some limitations, primarily the small number of patients who underwent all three examinations and its retrospective design, which may lead to less detailed data and a potential risk of verification bias, as the surgeon had access to all imaging results. However, no patients were excluded due to missing data, reinforcing the contribution of our findings to the existing evidence that ultrasound and MRI serve complementary roles in evaluating rotator cuff injuries in our setting.

## Conclusions

This study reaffirms that both ultrasound and MRI are effective tools for evaluating rotator cuff injuries, particularly in detecting complete supraspinatus tears, where both modalities demonstrated high sensitivity. However, diagnostic performance for partial tears remained limited, showing low sensitivity but good specificity. MRI showed superior accuracy in subscapularis tendon evaluation, likely reflecting ultrasound’s lower sensitivity and higher interobserver variability.

Importantly, our study contributes novel data by directly correlating imaging findings from both ultrasound and MRI with arthroscopic confirmation in the same patient cohort. This head-to-head comparison in a real-world clinical setting adds clarity to their respective diagnostic strengths and limitations. Moreover, the inclusion of bilateral lesions and the absence of missing data enhance the internal validity of our results. Despite some differences in performance, ultrasound remains a cost-effective and accessible option, especially for supraspinatus assessment. Ultimately, ultrasound and MRI play complementary roles, and selection should be tailored to clinical context and resource availability.
